# Establishment of an interdisciplinary board for bone and joint infections

**DOI:** 10.1007/s15010-021-01676-9

**Published:** 2021-08-02

**Authors:** Christina Otto-Lambertz, Ayla Yagdiran, Kirsten Schmidt-Hellerau, Charlotte Meyer-Schwickerath, Peer Eysel, Norma Jung

**Affiliations:** 1 Department of Orthopedics and Trauma Surgery, Medical Faculty, University Hospital of Cologne, University of Cologne, Cologne, Germany; 2 Department I of Internal Medicine, Medical Faculty, University Hospital of Cologne, University of Cologne, Cologne, Germany

**Keywords:** Bone and joint infection, Interdisciplinary board, Periprosthetic joint infection, Vertebral osteomyelitis, Osteomyelitis, Foreign material-associated infection

## Abstract

**Purpose:**

The incidence of bone and joint infections is increasing while their treatment remains a challenge. Although guidelines and recommendations exist, evidence is often lacking and treatment complicated by complex clinical presentations and therapeutic options. Interdisciplinary boards shown to improve management of other diseases, seem potentially helpful. We describe the establishment of an osteomyelitis board to show the existing demand for such a platform.

**Methods:**

All patients discussed in the board for bone and joint infections between October 2014 and September 2020 were included in this retrospective study. Data were extracted from patient records and analyzed descriptively.

**Results:**

A total of 851 requests related to 563 patients were discussed in the board during the study period. After a run-in period of 3 years, a stable number of cases (> 170/year) were discussed, submitted by nearly all hospital departments (22 of 25). Recommendations were mainly related to antibiotic treatment (43%) and to diagnostics (24%). Periprosthetic joint infections were the most frequent entity (33%), followed by native vertebral osteomyelitis and other osteomyelitis. In 3% of requests, suspected infection could be excluded, in 7% further diagnostics were recommended to confirm or rule out infection.

**Conclusions:**

A multidisciplinary board for bone and joint infections was successfully established, potentially serving as a template for further boards. Recommendations were mainly related to antibiotic treatment and further diagnostics, highlighting the need for interdisciplinary discussion to individualize and optimize treatment plans based on guidelines. Further research in needed to evaluate impact on morbidity, mortality and costs.

## Introduction

The number of bone and joint infections is increasing [1, 2], which is thought to be due to an increase in predisposing factors (e.g. diabetes mellitus [3] and an aging population [4]), of medical invasive procedures and of surgical treatment of degenerative bone and joint changes and fractures, often including incorporation of foreign material.

Diagnosis and therapy of bone and joint infections are multi-faceted and complex. Infections include septic arthritis, osteomyelitis of the long bones and vertebral osteomyelitis as well as fracture-related infections, periprosthetic infections and other foreign material-associated bone infections. Diagnosing bone and joint infections is often difficult even though diagnostic criteria have been published in international guidelines [5–12]. Common diagnostic challenges are antibiotic treatment before proper diagnostics, lack of relevant samples (e.g. superficial swipes instead of bone specimen) and discrimination between relevant pathogens and contaminants.

The building blocks of treatment come from both internal medicine and surgical disciplines. Surgical therapy is complex and antiinfective therapy often of long duration. Therapeutic options are time- and resource-consuming [13] and approaches often not evidence based. In addition to surgical interventions such as classical debridement, interventional approaches such as vascular recanalization and interventional pathogen retrieval (e.g. CT-guided puncture) are often required. Therefore, several disciplines are involved in the treatment of complicated infections and communication between them often poses a challenge. In the context of oncological diseases, interdisciplinary boards have already been implemented for many years [14] and are an integral part of certified cancer centers [15]. In infectious diseases (ID), interdisciplinary discussion in form of an endocarditis board has been shown to lead to optimization of treatment processes. As shown by Camou et al., a weekly board meeting facilitates following official guidelines while adapting them to the individual circumstances, leading to improved clinical outcomes in the treatment of endocarditis [16].

Regarding this previous evidence, an interdisciplinary approach including orthopedic and trauma surgeons, ID specialists, radiologists and microbiologists seems desirable for the optimal management of complex bone and joint infections, but this is rarely described. In France, for example, it is mandatory that complex bone and joint infections are treated in specialized centers with regular interdisciplinary meetings [17–19].

To meet this assumed need, an interdisciplinary weekly board for bone and joint infections (osteomyelitis board = OMB) was implemented in a tertiary care hospital in Germany in 2014. The board consists of a weekly meeting of an ID specialist, orthopedic and trauma surgeon, microbiologist and radiologist.

The aim of this study was to describe the establishment of an interdisciplinary osteomyelitis board, the number and nature of requests to show the existing demand for such a platform and the type of recommendations made.

## Materials and methods

### Study design and setting

A weekly multidisciplinary OMB was implemented in a tertiary care hospital, a 1430 bed facility treating 58,400 in-patients per year (average 2014–2020). Patients with suspected or proven bone and/or joint infections could be registered for discussion via the electronic health record. All patients discussed in the OMB between October 2014 and September 2020 were included in our retrospective study.

### Data collection, variables and statistics

Data were extracted from the written request and recommendation forms of the OMB in the electronic health record, anonymized and entered using Microsoft Excel 2010 software.

Recorded were the number of OMB requests, number of requests per patient, requesting departments, age and sex of patients. Further, the content of requests and recommendations was recorded and assigned to different diagnostic and therapeutic categories for analysis. In case of more than one OMB consultation for the same patient during the same inpatient stay, all consultations were analyzed.

Types of infection were classified as periprosthetic joint infection (PJI), other implant-associated bone infection, native vertebral osteomyelitis, other types of osteomyelitis and septic arthritis. Infections associated with internal plate and/or screw fixation were subsumed under "other implant-associated bone infections".

Data were analyzed descriptively using Microsoft Excel 2010. For categorical variables, absolute numbers and proportions were analyzed, and for continuous variables median and interquartile ranges were created. Figures were created by GraphPadPrism 9 software.

## Results

A total of 851 requests related to 563 patients were discussed in the OMB during the survey period from October 2014 until September 2020. Bedside infectious disease (ID) consultations also took place in 323/563 patients in addition to the OMB within 1 week in cases of particular complexity especially when the primary focus was unknown and bloodstream infection occurred.

**Fig. 1:**
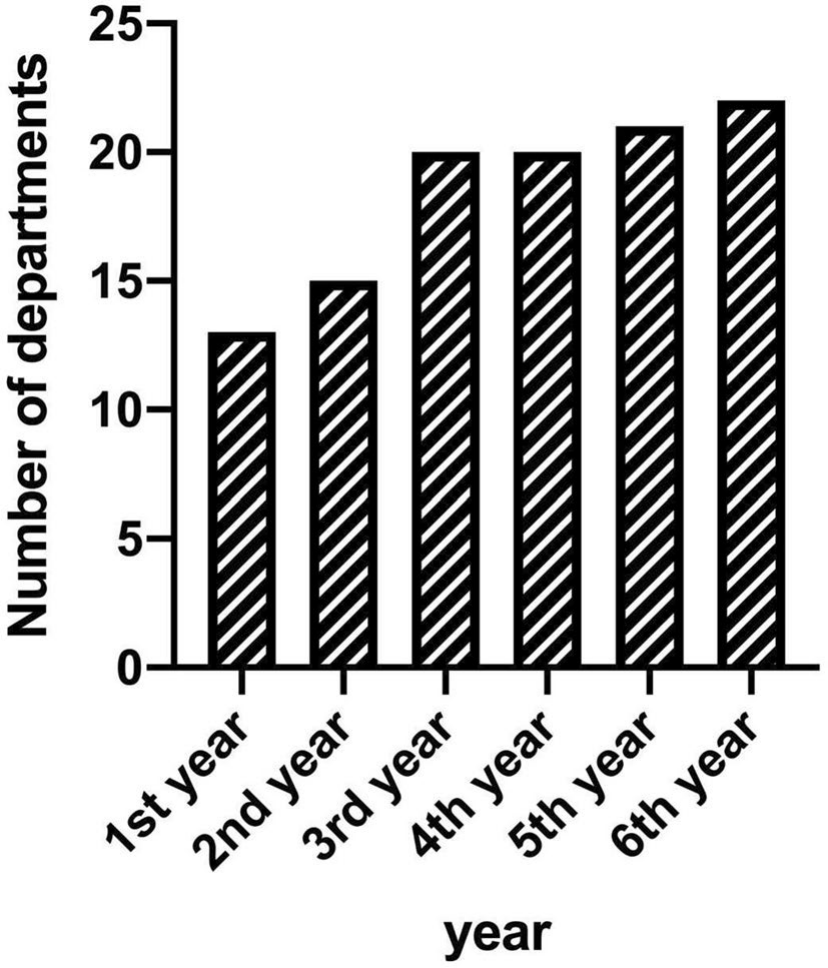
Development of the number of departments enrolling patients in the OMB (*OMB* osteomyelitis board). In the last year, cases were submitted by 22 of 25 departments involved in patient care at our university hospital

**Fig. 2:**
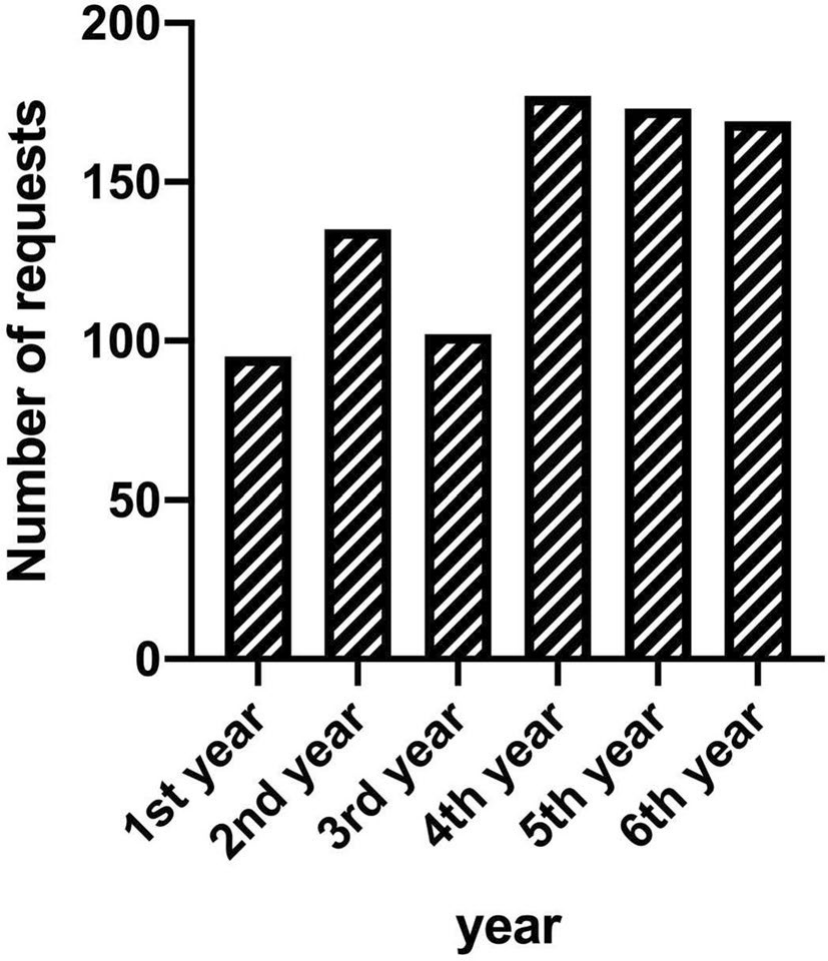
Development of requests for the OMB over the years (*OMB* osteomyelitis board). A continuous increase can be seen up to stable-high numbers of about 170 cases/year

The number of departments enrolling patients in the OMB increased from 13 different departments in the first year to 22 departments 6 years later (Fig. 1), corresponding to 88% (22 of 25) of departments involved in patient care at the university hospital. The total number of requests increased over a 3-year period until it reached a stable number of at least 170 requests per year (Fig. 2). One-third of patients (32%, *N* = 185) were discussed more than once, in few cases up to seven times (Table 1).

**Table 1:** Number of OMB requests and requesting departments; OMB: osteomyelitis board

Parameters	Number (%)
Number of patients	563 (100%)
Male	329 (58%)
Age [years] (median (IQR))	67 (53–76)
Number of patients discussed	
Once	378 (67%)
Twice	120 (21%)
Three times	42 (7%)
> = four times	23 (4%)
Total number of requests	851 (100%)
*Requesting department*	
Surgical	716 (84%)
Orthopaedics	545 (64%)
Trauma surgery	82 (10%)
Surgical outpatient department	47 (6%)
Anesthesia	15 (2%)
Vascular surgery	7 (1%)
Maxillofacial surgery	7(1%)
Other (Cardiac surgery, Neurosurgery, Urology, Ear, nose and throat surgery, Visceral surgery, Peadiatric surgery	13 (2%)
Non-surgical	135 (16%)
Hemato-oncology, Infectiology, Immunology	42 (5%)
General medicine, Nephrology, Rheumatology	38 (5%)
Paediatrics/paediatric oncology	17 (2%)
Cardiology, Pneumology	16 (2%)
Dermatology	15 (2%)
Other (Neurology, Endocrinology, Palliative care, Psychology)	7 (1%)

Requests mainly came from surgical (*N* = 716; 84%) and also from non-surgical departments (*N* = 135; 16%; see Table 1). Few requests (6%, *N* = 47) from surgical departments came from the outpatient clinics. Most requests were made by orthopedic surgery (*N* = 545; 64%), followed by trauma surgery (*N* = 82; 10%). Among the non-surgical departments, subspecialties of internal medicine [hemato-oncology/infectiology/immunology (*N* = 42; 5%); general medicine/nephrology/rheumatology (*N* = 38; 5%)] were the most frequent requestors (Table 1). Requests from surgical departments were more often related to antibiotic therapy only (46 versus 7%) while colleagues from non-surgical departments asked more often for advice related to antibiotic and surgical treatment (66 versus 38%).

The 851 requests led to a total of 1394 recommendations (see Table 2). Over 43% of these recommendations were related to antibiotic therapy. This included recommendations regarding duration of therapy in 25% and a change from intravenous to oral treatment in 17% of all recommendations. Almost a quarter of all recommendations (*n* = 334, 24%) were related to diagnostics, mainly imaging (including, e.g. MRI, CT and echocardiography), collection of material for microbiological diagnostics, consultations of other medical specialties and other examinations (for example, echocardiography or colonoscopy). In more than 16% (*n* = 222) of the recommendations, surgery was recommended (mainly surgical revisions with debridement with or without removal/change of foreign material). In only 2% of patients no change in the patient's therapeutic and diagnostic approach resulted from presentation to the OMB.

**Table 2:** Characteristic of OMB recommendations (OMB: osteomyelitis board)

Parameter	Number (%)
Total number of recommendations	1394 (100%)
Additional diagnostics	334 (24%)
Allergy testing in case of suspected antibiotic allergy	13 (1%)
Imaging	117 (8%)
MRI	66 (5%)
CT	15 (1%)
X-ray	20 (1%)
Other (PET-CT, FKDS, scinti, angio, sono)	12 (1%)
Collection of additional materials for microbiological examination*	68 (5%)
Blood culture	37 (3%)
Consultation	61 (4%)
Infectiology	26 (2%)
Orthopedics/trauma surgery	10 (1%)
Rheumatology	5 (0.4%)
Other examinations	70 (5%)
Echocardiography	34 (2%)
Colonoscopy	8 (1%)
Recommendations regarding antiinfective treatment	599 (43%)
Change from IV to PO	241 (17%)
Determination of treatment duration	343 (25%)
Change of drug	130 (9%)
Surgery (e.g. debridement, removal/change of foreign material)	222 (16%)
Intervention (e.g. interventional vascular recanalization, joint/pleura puncture, change of catheters)	18 (1%)
Follow up only (clinical/radiological)	31 (2%)
Maintain previous procedure	32 (2%)
No initiation of antiinfective treatment recommended (e.g. pathogen is evaluated as contaminant)	28 (2%)

*For each (revision) surgery the collection of deep samples for microbiological and pathological examination was recommended, if possible

Among the 563 patients, 47 (8%) presented with more than one focus of infection, mostly an additional bone or joint infection (see Table 3). Different infectious foci in one patient either occurred per continuitatem (e.g. osteomyelitis of tibia and soft tissue infection of lower leg) or via hematogenous dissemination (e.g. vertebral osteomyelitis and shoulder empyema). Bacteremia was detected in 19 patients, among these 6 patients suffered from endocarditis.

**Table 3:** Characterization of patients with more than one focus of infection, categorization according to the leading infection focus (*PPI *periprosthetic infection)

Number of patients with more than one focus of infection	47
Bilateral joint infection (native knee infection and PPI)	4
Vertebral osteomyelitis AND	
Shoulder empyema	1
Osteomyelitis of foot	1
Osteomyelitis of femur	1
Osteomyelitis of sternum AND sacrum	1
Empyema of shoulder AND soft tissue infection (M. psoas, M. pectoralis)	1
Hip empyema	2
Knee empyema	1
PPI of hip	2
PPI of knee	1
Periprosthetic infection of hip AND	
PPI of knee	
Suspected hip empyema	4
Osteomyelitis of foot AND	1
Empyema of shoulder	1
Osteomyelitis of symphyse AND	
Soft tissue infection of symphyse	5
Osteomyelitis of tibia AND	
Soft tissue infection of lower leg	1
Empyema of hip AND	
Bilateral shoulder empyema AND soft tissue infection (back muscles)	2
Vertebral osteomyelitis AND abscesses (lung and muscles) AND suspected infection of thrombosis femoral	1
osteomyelitis of sternum	1
osteomyelitis of sternum AND mediastinitis	1
Empyema of knee AND	
Suspected infection of spine	3
Soft tissue infection of knee	1
Osteomyelitis of shoulder AND osteomyelitis of clavicula AND soft tissue infections (abscess of lung, endophtalmitis, suspected septical embolie)	1
Infection post-surgery (tibia) AND	
Knee empyema	1
Infection post-surgery femur	1
Suspected infection of spine AND knee joint	2
Suspected infection of hip joint AND spine	1
Suspected infection of tibia AND femur	1
Foreign material infection of the spine AND	
Septic herd encephalitis AND septic arthritis of both feet	1
Vertebral osteomyeltis (another level)	2

**Fig. 3:**
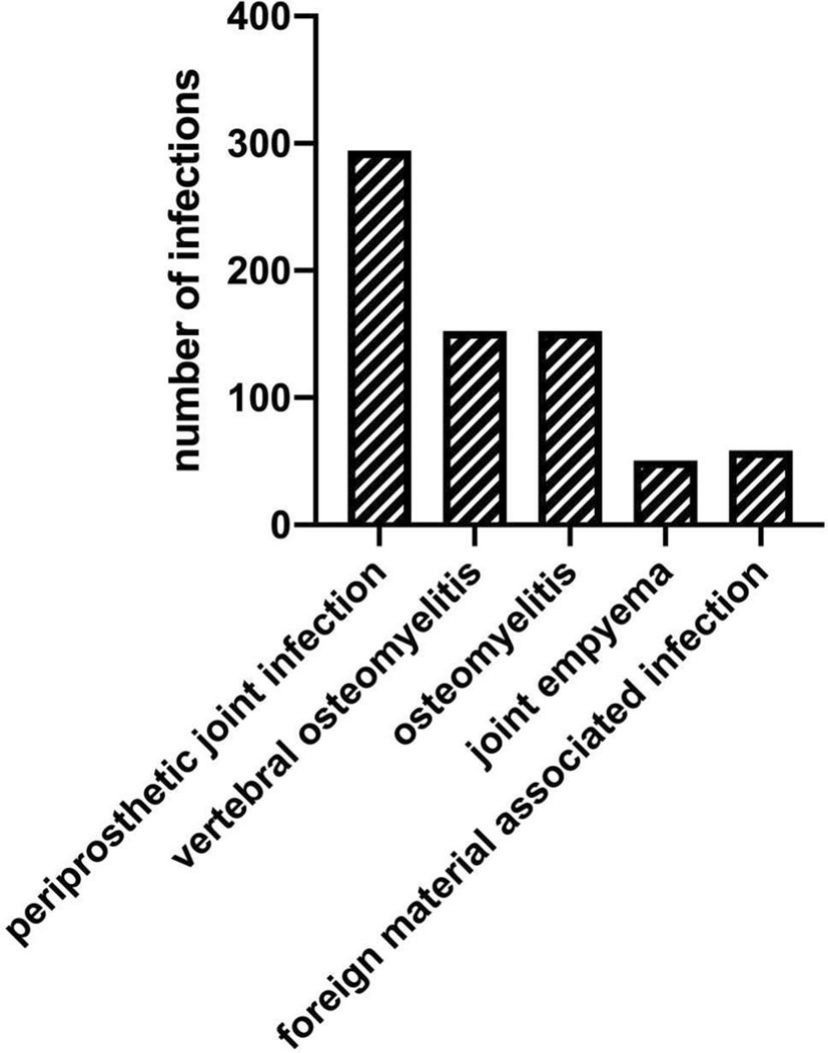
Classification of the 706 bone- and joint-associated infections discussed at the OMB (*OMB* osteomyelitis board). Periprosthetic infections were counted as a separate entity, infections associated with internal plate and/or screw fixation were subsumed under "foreign material-associated infections"

Over all, 900 suspected foci of infection were discussed. Of these foci, 28 were classified as non-infectious without indication for antiinfective treatment (in 12/28 a detected pathogen was assessed to be an irrelevant contaminant, in 9/28 only clinical follow-up, 7/28 further investigation to assess differential diagnosis). A further 67 of the 900 suspected foci of infection were evaluated to be possibly infectious, and additional diagnostics were recommended to either confirm or rule out infection. Periprosthetic joint infection (*N* = 294) was most frequent among the confirmed bone and joint-associated foci (*N* = 706/900) (Fig. 3) followed by surgical wound infections (70/900) and by other soft tissue infections (29/900).

61 (9%) patients presented to the OMB for interdisciplinary evaluation had a history of malignant tumors and 80 (12%) of trauma in the area of infection.

## Discussion

We describe the implementation, utilization and activities of an interdisciplinary board for bone and joint infections. The number of requests to the board shows a perceived need to discuss these cases, while an analysis of the recommendations made by the board showed that in almost all cases interdisciplinary discussion led to recommendations regarding additional diagnostics and/or changes in treatment, suggesting a potential impact on outcomes.

After an initial run-in period of 3 years, which is possibly due to an increasing awareness of the board in the different departments, the OMB seems well accepted with a now continuously high number of requests submitted by almost all departments of the university hospital. A similar run-in period of up to 5 years was described by Rieg et al. for the initialization of an infectious disease consultation service at four university hospitals in Germany [20]. The continuously high numbers of request from almost all departments of our tertiary care facility show an existing demand for such a platform.

Diagnoses discussed in the OMB were mainly periprosthetic infections and osteomyelitis. Due to a significant increase of artificial joint implantations in recent years, a high number of periprosthetic infections were expected, as this is a typical complication of artificial joint implantation [21, 22]. Vertebral osteomyelitis, known to have an increasing incidence over the last years [23, 24], was the second most frequently discussed diagnosis along with osteomyelitis of other bones [25]. The acceptance and regular use of the OMB for these types of infection indicate a perceived need for an interdisciplinary discussion of these complex cases.

The high number of board recommendations regarding changes in antibiotic treatment and/or treatment duration as well as at the number of suspected infectious foci that were evaluated to be non-infectious (*N* = 28/900) hint at a relevant impact of the OMB on quality and quantity of antibiotic treatment. This is in line with a survey from Switzerland investigating the activity and impact of an ID specialist in a septic orthopedic unit on antibiotic use and costs [26]. Here, a substantial decrease in antibiotic use and costs was shown without a concurrent increase of recurrent infections.

Bedside ID consultations took place in addition to the OMB presentations in cases of particular complexity. Extensive data are available for the benefit of ID consultation services especially in the context of *S. aureus* bloodstream infection and also from a growing number of studies showing a substantial role in surgical and medical wards [20, 27–31]. Of note, phone consultations do not seem to be equivalent to consultations with bedside examination of the patients. In a retrospective analysis, mortality for *S. aureus* bloodstream infection was almost double for phone consultations compared to bedside ID consultations [32]. Therefore, it seems unlikely that multidisciplinary board discussions for complex bone infections can substitute bedside ID consultation entirely, but multidisciplinary board discussion seems to be a reasonable complementary tool with first data suggesting a benefit. For vertebral osteomyelitis, Ntalos et al. preformed a retrospective pre–post intervention study comparing a single discipline approach with a weekly multidisciplinary infections conference [33]. Here, multidisciplinary conference led to significant changes in antiinfective and surgical treatment and reduced days of antibiotic treatment, while no differences were detected for in-hospital complications or total in-hospital stay.

Our study has several limitations. As it was performed in one German tertiary care centre, our results cannot be easily generalized and need to be validated for primary care hospitals and other countries. Due to our study design which focused on the implementation and activities of the OMB, adherence to recommendations and the impact on outcome parameters as morbidity, mortality and cost savings could not be evaluated. Nevertheless, a main strength of our study is the detailed evaluation of the establishment of an interdisciplinary board for bone and joint infections showing an existing demand and offering precise descriptions of the activities, these key figures that may inform both planning of similar boards in other settings and of studies evaluating such boards.

## Conclusion

Multidisciplinary boards have shown to be beneficial in the treatment of a number of diseases, but not yet for bone and joint infections, even though these often require the expertise of several specialties. A newly established board for bone and joint infections in an university hospital showed an increasing number of requests from an increasing number of departments, showing a perceived need for interdisciplinary discussion of these often challenging cases. Board recommendations were mainly related to changes in antibiotic treatment and additional diagnostics, hinting at a potential impact of such discussion on individual treatment, while formal outcome evaluation is pending.

## Data Availability

The original data are held by the corresponding author and can be viewed there at any time.
